# Molecular Markers of Tumor Progression in Melanoma

**DOI:** 10.2174/138920209788488526

**Published:** 2009-06

**Authors:** Joshua Rother, Dan Jones

**Affiliations:** Division of Pathology and Laboratory Medicine, The University of Texas M. D. Anderson Cancer Center, 1515 Holcombe Boulevard, Houston, TX 77030, USA

**Keywords:** Melanoma, genetic progression, tumor suppressors, cell cycle, alkylating agents, mitotic spindle, mitotic spindle poisons.

## Abstract

Malignant melanoma represents one of the most aggressive malignancies but outcome is highly variable with early tumor lesions having an excellent prognosis following resection. We review here the data on identification of genes involved in the progression of melanoma as a result of expression array studies, genomic profiling, and genetic models. We focus on the role of tumor suppressors involved in cell cycle function, DNA repair, and genome maintenance. Highlighted are the roles of loss of p16 in promoting neoplasia in cooperation with deregulated MAPK signaling, and the role of loss of the RASSF1A protein in promoting chromosomal instability. The interactions between point mutation in growth signaling molecules and epigenetic changes in genes involved in DNA repair and cell division are discussed.

## I. HETEROGENEITY IN MELANOMA: CLINICAL PRESENTATION AND OUTCOMES

Melanoma arises from malignant transformation of melanocytes and has an aggressive course once the tumor has spread beyond the superficial skin. However, like all tumor types there is considerable heterogeneity in outcome and molecular pathogenesis. Clinically distinct patterns of melanoma include acral lentiginous (AL) presenting in the distal extremities, and superficial spreading (SS), lentigo maligna, and nodular types. Almost all histologic and clinical patterns of melanoma are increased in patients with a history of heavy sun exposure, particularly discrete serious sunburn episodes but other risk factors are poorly understood. Mucosal and soft tissue presentations of melanoma while rare appear to have a distinct pathogenesis. There are also a variety of different histologic appearances in melanoma including the typical epithelioid forms, as well as desmoplastic (spindle cell) and anaplastic variants. Approximately 5-10% of melanomas have a strong familial link, and the molecular defects in most of these cases involve cell cycle regulators, particularly cyclin-dependent kinases (CDKs) and the CDK inhibitor p16 (CDKN2A) in their molecular pathogenesis [1-5].

## II. THERAPEUTIC OPTIONS FOR MELANOMA

Outcome in melanoma is highly dependent on the depth of invasion seen in the primary lesion. The predominant therapeutic modality in melanoma remains surgical resection with adequate margins. However, patients with residual disease after resection, with lymph node metastases, or with distant metastatic spread usually receive adjuvant therapy [6]. Radiation therapy is employed for symptomatic relief of brain and visceral metastases that cannot be resected. Advanced melanoma is highly resistant to most forms of chemotherapy and response rates to DTIC (dacarbazine) or other alkylating agents such as temozolomide (TMZ) [7], carmustine and lomustine in combination with spindle poisons such as vincristine are seen in approximately 10 to 20% [8-11]. Biologic treatments are also employed for metastatic melanoma, with trials using interferon-alpha and interleukin-2 generally showing responses in 10 to 20% of patients [12-14]. However, these treatment strategies have not yet been optimized due to a lack of biomarker predictors of response to either approach.

## III. MOLECULAR MARKERS OF DYSREGULATED GROWTH FACTOR SIGNALING AND THE USE OF TYROSINE KINASE INHIBITORS

**Fig. (1):**
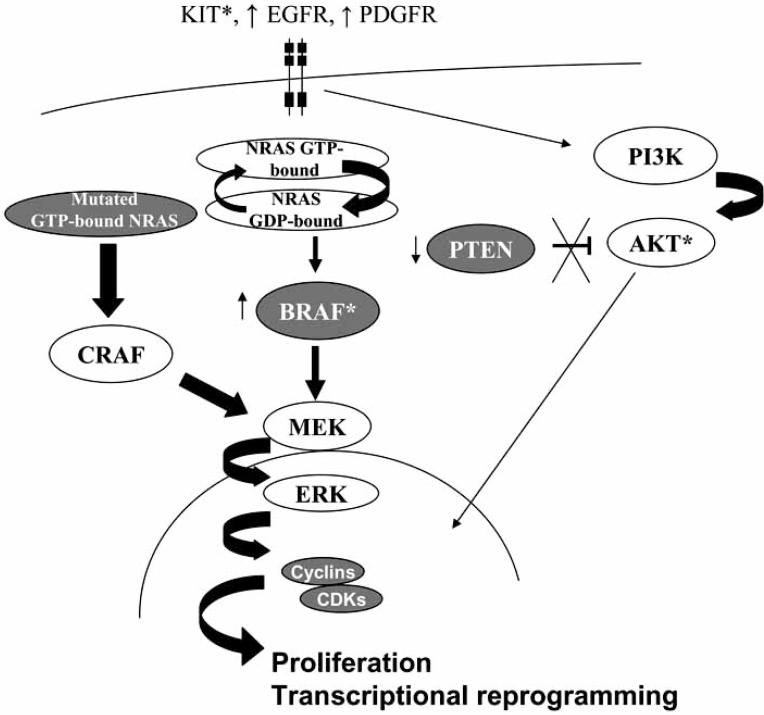
**Receptor tyrosine kinase (RTK) pathway dysregulation in melanoma pathogenesis**. Genetic alterations seen in different subsets of melanoma include point mutation (*) of the RTK KIT, genomic amplification (↑) of EGFR and PDGRFA, point mutation of NRAS and BRAF, complete loss of PTEN expression (↓), and rarely point mutation of AKT1 and AKT3 (*).

Many of the best characterized somatic mutation and epigenetic changes in melanoma involve the receptor tyrosine kinase (RTK) signaling pathways, which are particularly implicated in sun-exposed (cutaneous) cases [15]. These genetic alterations include BRAF, NRAS, KIT and (rarely) AKT1/3 point mutation, EGFR and PDGRA genomic amplification, and epigenetic silencing or mutation of the tumor suppressors PTEN and FGFR2 (Fig. **1**).

BRAF is a serine/threonine kinase that signals downstream of RTKs and ras proteins. 30-70% of melanomas show BRAF point mutations which alter the autoregulatory activation of the kinase, of which the V600E mutation is by far the most common. BRAF mutations are most common in the nodular and SS types, and rare in AL (5-10% of cases) and non-cutaneous melanomas [16-18] (Table **1**). BRAF mutation correlates with distinct histopathologic features, such as intraepidermal melanoma nest formation and a larger rounder border of the tumor with surrounding skin, suggesting surrogate markers can be used to select cases for molecular testing [19]. BRAF mutations also arise more commonly in patients with younger age at presentation and lymph node metastasis (rather than satellite tumors or visceral metastasis) [19]. However, benign nevi show similar or higher rates of the V600E BRAF mutation [20, 21], and cell line and transgenic mouse models of melanoma do not clearly demonstrate the transforming power of this mutation. Germline BRAF mutations do not occur in melanoma [22].

NRAS is a GTPase protein which functions to integrate signals from multiple RTKs. 10-20% of melanoma have point mutations in codon 12, 13 or 61 of NRAS, which are almost always mutually exclusive with BRAF mutation [15, 17, 18, 23]. This is possibly because NRAS-mutated melanoma may bypass BRAF and signal through CRAF [24]. NRAS mutations are rarely found in benign acquired nevi (although seen in congenital nevi) [25], arise later in melanoma development, and can produce melanoma in certain animal models and are thus are more clearly implicated in oncogenesis than BRAF mutations.

KIT is a RTK that is essential for normal neural crest/melanocyte differentiation. Activating KIT mutations are typically seen in mucosal and AL melanoma subtypes (5-20% of cases) but not in cases arising from chronic sun damage. Point mutations frequently occur in KIT exons 11, 13 and 17 [26]. The L576P mutation is most common (comprising 50% of mutations). KIT gene amplification also occurs [21, 27]. KIT mutations are mutually exclusive with BRAF and NRAS, and may identify a subset of melanoma that preferentially respond to the KIT inhibitors such as imatinib (Gleevec) [28, 29] or sorafenib [30, 31]. There are also regulatory changes in KIT expression during melanoma progression. For example, the highest levels of KIT expression are seen at the leading edge of tissue invasion which may indicate a role for dynamic RTK activation in metastasis [26].

EGFR is a RTK implicated in normal epithelial and melanocyte maturation. It is often overexpressed by gene amplification (usually whole gain of chromosome 7) in metastatic melanomas [32], but the prognostic impact of detection of gene amplification remains unresolved. Rare cases of melanoma (often of desmoplastic type) may show activating EGFR mutations. The RTKs platelet-derived growth factor receptor (PDGR)-alpha and PDGFR-beta are also highly expressed in melanocytic lesions [33]. Genomic profiling has revealed that AL and mucosal melanomas can have chromosomal amplification at chromosome 4q11spanning the PDFGRA locus [34, 35] that may contribute to increased PDGF signaling [36]. Finally, dominant-negative FGFR2 mutations have been reported in approximately 10% of nodular melanomas [37]. FGFR2 is a RTK that may mediate growth arrest in melanoma through interactions with stroma so its inactivation may promote tissue invasion.

PTEN is a phosphatase that regulates the activation of the serine/theronine kinases AKT1/2/3, which are global regulators of cell proliferation. PTEN is regulated as a tumor suppressor with complete PTEN loss (usually accompanied by genomic deletion) seen in 20-25% of melanomas (including those with BRAF mutation) [38-42], and is highly associated with uniform high-level AKT activation [43]. PTEN loss and concomitant AKT activation are both usually demonstrated by immunohistochemistry, with an anti-phosphoprotein antibody against an activation site on AKT (pS473) [40, 43]. Rare activation mutations in the AKT1 or AKT3 isoforms have been found in sun-exposed melanoma subtypes [44], and AKT overexpression may be associated with melanoma growth *in situ* [45, 46].

Variations in the incidence of different RTK pathway mutations in different geographic populations are evident [47], as well as variations in risk related to polymorphisms in other susceptibility loci [48-50]. Since most advanced-stage melanomas are resistant to existing adjuvant therapies, kinase inhibitors (KIs) have been tried in melanomas that demonstrate mutational activation of the kinases above. In single case reports or in small series, KIs have shown promising short-term responses that generally correlate with the presence of targetable RTK mutation in the tumor. For example, administration of sorafenib (a KI with activity against RAF, PDGFR, VEGF, and KIT) along with carboplatin and paclitaxel in a phase II trial has led to a partial response rate of 26% [51]. Results on the use of imatinib (Gleevec), a KI with high activity against KIT and PDGFR, have been disappointing with responses possibly correlated with either KIT mutation or high-level KIT protein expression (e.g. ≥75% of tumor cells) [52].

## IV. MARKERS OF MELANOMA PROGRESSION IDENTIFIED BY GENE EXPRESSION STUDIES

Gene expression changes that occur during tumor progression can be due to chromosomal gains/losses resulting from cell cycle alterations (discussed below), activating mutations in pathways that modulate transcription factors (e.g. the RTK pathway mutation), or by epigenetic regulation. Melanoma at sun-exposed sites may more frequently demonstrate (UV-induced) genetic mutation whereas melanomas arising at non-sun-exposed sites may more frequently utilize epigenetic regulation but overlapping patterns are clearly seen.

This role of epigenetic regulation is clearly highlighted by silencing of multiple different tumor suppressor genes during melanoma progression. For example, the cell cycle regulator p16 is frequently silenced by promoter DNA CpG methylation (e.g. 32% of uveal melanoma) [53], as is the APC gene which regulates Wnt signaling in 10-20% of cases [54], and the kinase regulator RASSF1 in up to 50% of cases [55]. The DNA repair gene MGMT (O-6-methylguanine-DNA-methyltransferase) is silenced in approximately 20% of melanoma [56], and its inactivation corresponds with declines in the ability to repair DNA which may promote mutagenesis and potentiate the response to DNA-damaging chemotherapy [57].

Microarray gene expression profiling of primary and progressed melanoma and melanoma cell lines have revealed many of the coordinated changes in gene expression that correlate with clinical stage [36, 56, 58-65]. For example, early-stage melanomas often express high levels of the immune modulator CD24 and the transcription factor GATA3, whereas progressed melanomas exhibit upregulation of the melanoma antigen family A (MAGE) antigens of unknown function, and cell cycle regulators such as CDK2 [36]. The commonalities arising from these GEP studies of melanoma progression (Table **2**) highlight several fundamental patterns of transcriptional dysregulation that may prove useful in individualizing therapy response and in developing novel treatment strategies.

## V. G1-S CHECKPOINT ALTERATIONS IN MELANOMA PROGRESSION

**Fig. (2):**
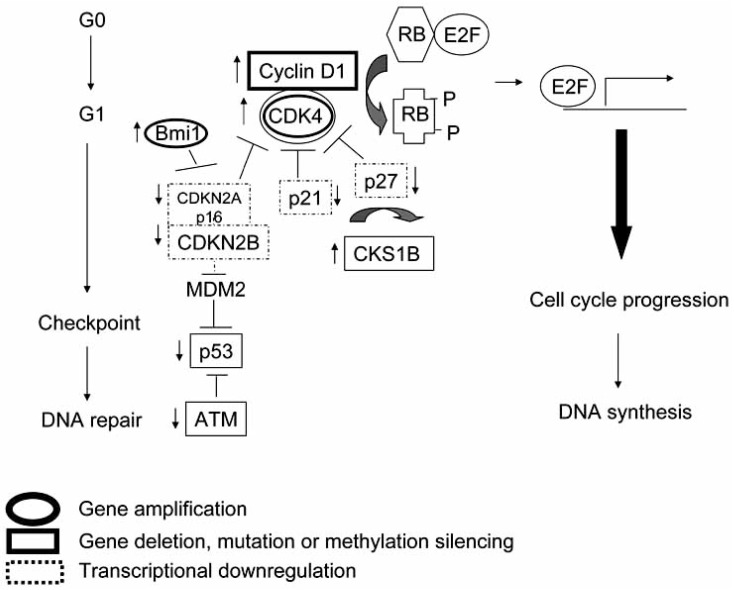
**Cell cycle dysregulation in melanoma progression**. Complex patterns of transcriptional and epigenetic regulation of the cyclin-dependent kinases (CDKs), CDK inhibitors and the p53 axis have been demonstrated in microarray genomic and expression studies of melanoma progression. These include downregulation (↓) of inhibitors of CDK by DNA methylation silencing, deletion and transcriptional networks, and genomic amplification and microRNA regulation of CDKs and cyclins.

Genetic studies [4], genomic profiling [34], and GEP have all highlighted the multiple overlapping genetic and epigenetic alterations in the proteins regulating the G1-S transition in the cell cycle (Fig. **2**). Among the 5-10% of melanomas with strong familial linkage or inherited germline defects, mutations in p16 (CDKN2A) [4, 5] and CDK4 [3] are common findings, as is loss of p27 [66, 67] and p16 [68, 69] expression during progression of sporadic melanomas. Indeed, loss of p16 appears to be common to most melanoma subtypes including superficial spreading, mucosal and nodular cases [38, 68-70]. Secondary genetic changes in melanoma also frequently involve the same genes, evidenced by the frequent genomic deletions at chr 12q14 spanning the CDK4 loci in AL melanoma [67], and chr 17p13 loss at the TP53 locus in chronic sun-damage melanomas [34].

Multiple abnormalities in the G1-S proteins can occur simultaneously in the same melanoma, and occur in tandem with RTK alterations outlined above. All of these changes would be expected to mediate rapid progression through G1-S leading to propagation of unrepaired DNA errors. Although these effects are deleterious in terms of cancer progression, they may predict responsiveness to DNA-damaging chemotherapy such as alkylating agents, as discussed below.

## VI. G2-M ALTERATIONS IN MELANOMA PROGRESSION

Another major cellular pathway that becomes dysregulated in melanoma progression is the G2-M mitotic transition. This stage of the cell cycle is regulated by a dynamic multi-protein spindle checkpoint complex that assures adequate centrosomal function and accurate chromosomal segregation. Upon activation, the centrosome divides to form spindle poles which function to guide chromosomal segregation. The progression from G2 to M is initiated at the centrosome by CDK1 and cyclin B if the checkpoint is adequately functioning. Spindle function can be negatively regulated by p21 and p27 which we have previously discussed as G1-S regulators that are frequently dysregulated in melanoma [71]. Other regulators of centrosomal/spindle pole function include the Aurora kinases and RASSF1A [71, 72], both of which been shown by us or others to be dysregulated during melanoma progression.

**Fig. (3):**
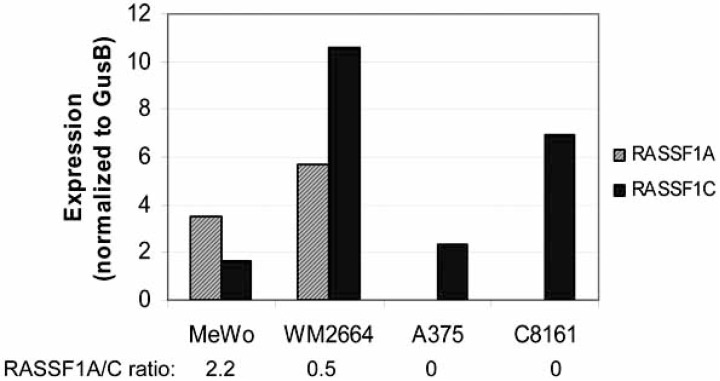
**Variations in RASSF1 isoforms in melanoma cell lines**. A. The MeWo line, established from a lymph node melanoma metastasis, shows higher RASS1A levels compared to RASSF1C. WM-2664, established from a cutaneous melanoma, shows higher RASSFC than RASSF1A. Lines A375 and C8161 lack RASSF1A expression due to promoter methylation silencing. Studies were performed by TaqMan reverse transcription (RQ)-PCR using a RASSF1A-specific primer-probe set (Applied Biosystems, Foster City, CA) with normalization to GUSB transcript levels.

The RASSF1 gene, located at chr 3p21.3, has several different splice isoforms that encode proteins with SARAH, ras-association and diacylglycerol-binding domains. Although their functions have not been completely elucidated, RASSF1 proteins appear to bind and stabilize a number of different kinase complexes involved in apoptosis, proliferation, and genome maintenance [73, 74]. There are at least 5 RASSF1 splice isoforms transcribed from different promoters but we find that only the A and C isoforms are expressed in melanomas. We and others have shown that the RASSF1A splice isoform is differentially silenced by CpG DNA methylation during melanoma progression in 20-50% of primary tumors and in established melanoma cells lines [55, 75]. This silencing results in an imbalance between the amount of RASSF1A and the ubiquitously expressed RASSF1C isoforms (Fig. **3**).

**Fig. (4):**
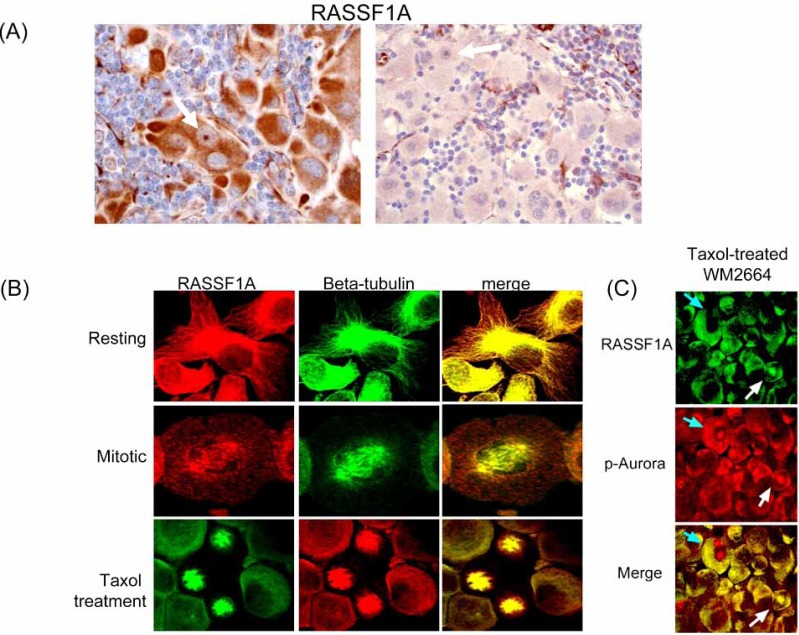
**Variable RASSF1A expression in melanoma**. (**A**) High level RASSF1A expression in the cytoplasm of a melanoma metastatic to lymph nodes is contrasted with near absence of expression in another progressed melanoma. White arrows highlight the nuclear localization of RASSF1A seen in cycling cells. Immunohistochemical staining was performed on formalin-fixed paraffin-embedded tumor sections using a mouse monoclonal antibody (eB114-10H1. eBioscience, San Diego, CA) and the ABC avidin-biotin detection method. Activated endothelium within each tissue serves as a positive control. (**B**) RASSF1A in non-dividing cells is present in the cytoplasm in association with the actin-tubulin cytoskeleton. During the later stages of mitosis, RASSF1A transiently colocalizes with tubulin and other spindle components at the spindle poles. Use of mitotic spindle inhibitor (paclitaxel) results in trapping of RASSF1A in stalled mitotic spindle complexes. Confocal microscopy performed with RASSF1A polyclonal antisera (N-12, Santa Cruz Biotechnology, Santa Cruz, CA), and a beta-tubulin mouse monoclonal antibody (clone DM1A, Sigma). (**C**) In melanoma cell lines, RASSF1A and Aurora kinases colocalize at the mitotic spindle in a subset of cells. Confocal microscopy was performed using a pan-phospho-Aurora antisera (Thr288A/Thr232B/ Thr198C, Cell Signaling Technology, Beverly, MA) and a RASSF1A mouse monoclonal antibody (eB114-10H1, eBioscience).

The best characterized function for RASSF1A is in complex stabilization of the mitotic spindle during one phase of the G2-S transition [72, 76]. But we have noted that differences in the levels of RASSF1A (due to varying levels of CpG methylation of its promoter) in melanoma cells leads to altered cellular localization patterns and likely different functions. For example, melanomas with very low RASSF1A expression show discrete nuclear positivity in only rare cycling tumor cells, whereas tumors with high RASSF1A expression show preferential cytoplasmic, membrane and nuclear localization patterns depending on tumor type (Fig. **4A** and not shown).

During cell division in melanoma, RASSF1A shifts from its predominant localization with the microtubles in the cytoplasm to discrete locations within the mitotic spindle (Fig. **4B**). These shifts are transient and dependent on the recruitment of other spindle components such as Aurora kinases (Fig. **4C**). Melanoma cells treated with spindle toxins such as paclitaxel or vinblastine show trapping of RASSF1A in the altered mitotic spindle. Tumor cells with diminished RASSF1A have a greater tendency to develop chromosomal aberrations [77, 78].

## VII. IDENTIFYING WHICH MELANOMAS MIGHT BENEFIT FROM CHEMOTHERAPY

Although profiling of growth factor pathway alterations may be useful in selecting patients for KI therapy, NRAS and BRAF mutation status have shown no or limited correlation with response to chemotherapy or immunotherapy. There is as yet too limited data on the correlative responses of melanomas with FGFR2, PTEN/AKT or KIT mutations. Therefore most studies have focused on identifying predictors of chemotherapeutic response.

The drugs typically used to treat melanoma include carboplatin and cisplatin, alkylating agents, and mitotic spindle poisons such as vinblastine and paclitaxel. Resistance to cisplatin in melanoma may be related to sequestration of the drug in melanosomes [79, 80], and resistance to alkylating agents may be mediated by expression of MGMT, which opposes their action [81]. However, since therapeutic activity of alkylating agents and DNA-damaging agents require tumor cell division, melanomas with a higher proliferative rate or those with genetic alterations in checkpoint function may be more likely to respond [82].

Since the spindle poisons (paclitaxel, vincristine, or vinblastine) are typically components of most multi-agent chemotherapy regimens, identification of predictors of response to this class of agents would be clinically useful. These drugs block cell division by interfering with microtubule function essential for chromosomal segregation and cytokinesis. Since abnormalities in mitotic regulators such as RASSF1A and Aurora kinases are common in melanoma they represent obvious candidate biomarkers. Indeed, in advanced stage melanoma, RASSF1A appears to correlate to some degree with response to chemotherapy. As a result, profiling of the activation state or the degree of mitotic spindle dysfunction using these markers shows promise in identifying those patients who would benefit most from spindle toxins. Additionally, strategies to restore loss of expression of spindle checkpoint proteins such as RASSF1A by use of demethylating agents (or more targeted methods) may be useful in reversing genetic instability associated with tumor progression.

## Figures and Tables

**Table 1.** Incidence of Growth Factor Mutations in Different Melanoma Subtypes

Site of Melanocytic Lesions	Incidence of RTK Mutations/Amplification	Incident of Genetic/Epigenetic Cell Cycle Alterations	References
Chronic sun-damaged sites	BRAF: 10% NRAS: 20% KIT mut/amp: 28%	CDKN2A/p16 loss: 88%	[17, 26, 70]
Nodular sun-exposed sites	BRAF: 36.3% NRAS: 54.5% PTEN loss of expression: 7%	CDKN2A/p16 loss: 78.9% p27KIP1 LOH: 38.9%	[18, 37, 40, 67, 69]
Acral melanoma	PDGFRA amp: 18% KIT mut/amp: 36% FGFR2 mutation: 4%	CDK4 amp: 23% CCND1 (cyclin D1) amp: 23% PDGFRA amp: 18%	[26, 34, 37]
Mucosal melanoma	KIT mut/amp: 53.3% FGFR2 mutation: 6%	CDKN2A/p16 LOH: 33%	[16, 26, 37, 68]
Uveal melanoma	PTEN LOH: 39.5% PTEN loss of expression: 16%	CDKN2A/p16 methylation: 32%	[39, 53]
Congenital nevi	NRAS: 81.3%		[25]
Sporadic nevi	BRAF: 20%		[20]
Inherited melanoma syndromes		CDKN2A/p16 mutation: 70% CDK4 amp: (infrequent)	[1-4, 58]

Abbreviations: mut: point mutation; amp: genomic amplification; LOH: loss of heterozygosity (genomic deletion).

**Table 2.** Genes Involved in Melanoma Progression Identified by Gene Expression Profiling

Gene	Function	Fold-Change	Comparison Group	References
**Upregulated**				
BIRC5	Component of chromosome passenger complex that ensures chromosome alignment/segregation	↑3-5X	primary → metastasis primary → metastasis	[36, 64]
BUB	Mitotic kinase that functions in spindle checkpoint function	↑4-11X	primary → metastasis nevi → melanoma	[36, 59]
CDK2	Kinase that regulates the G1-S transition	↑3-9X	primary → metastasis nevi → melanoma	[36] [65]
CHEK1	Mitotic kinase that phosphorylates cdc25 at G2-M transition	nr	nevi → melanoma blood of metastatic cases	[60, 62]
CCNA2 (cyclin A)	Binds and activates CDC2 and CDK2 at the G1-S and G2-M transition	nr	nevi → melanoma	[60, 63]
MAGEA1	Mediator of transformation through extracellular/adhesion signaling	↑25X	primary → metastasis primary → metastasis	[36, 61]
MAGEA2	As above	↑31X	primary → metastasis primary → metastasis	[36, 61]
**Downregulated**				
MAP4	Microtubule binding protein stabilizing the cyclin B/CDC2 kinase mitotic complex	↓20X	nevi → melanoma	[36]
CDKN2A/p16	Cyclin-dependent kinase inhibitor that regulates G1-S transition	nr	primary → metastasis primary → metastasis	[58] [83]
CDKN1B/p27	Inhibitor of cyclin E-CDK2 and cyclin D-CDK4 complexes at G1-S transition	nr	primary → metastasis primary → metastasis	[67] [58]
SFN	Inhibitor of p53 function at G2-M transition	↓24X	primary → metastasis primary → metastasis	[36, 61]
FGFR3	RTK stromal signals/differentiation	↓8X	primary → metastasis	[36, 61]
